# SpaceOAR© hydrogel rectal dose reduction prediction model: a decision support tool

**DOI:** 10.1002/acm2.12860

**Published:** 2020-04-30

**Authors:** Owen Paetkau, Isabelle M. Gagne, Abraham Alexander

**Affiliations:** ^1^ Department of Physics and Astronomy University of Victoria Victoria BC Canada; ^2^ Department of Medical Physics BC Cancer – Victoria Victoria BC Canada; ^3^ Department of Radiation Oncology BC Cancer – Victoria Victoria BC Canada; ^4^ Department of Surgery University of British Columbia Vancouver BC Canada

**Keywords:** decision support tool, linear modeling, rectal dose, SpaceOAR hydrogel

## Abstract

Prostate cancer external beam radiation therapy can result in toxicity due to organ at risk (OAR) dose, potentially impairing quality of life. A polyethylene glycol‐based spacer, SpaceOAR© hydrogel (SOH), implanted between prostate gland and rectum may significantly reduce dose received by the rectum and hence risk of rectal toxicity. SOH implant is not equally effective in all patients. Determining patients in which the implant will offer most benefit, in terms of rectal dose reduction, allows for effective management of SOH resources. Several factors have been shown to be correlated with reduction in rectal dose including distance between rectum and planning treatment volume (PTV), volume of rectum in the PTV, and change in rectum volume pre‐ to post‐SOH. Several of these factors along with other pre‐SOH CT metrics were able to predict reduction in rectal dose associated with SOH implant. Rectal V55Gy metric, was selected as the dose level of interest in the context of 60 Gy in 20 fraction treatment plans. Models were produced to predict change in RV55Gy and pre‐SOH hydrogel RV55Gy. These models offered R‐squared between 0.81 and 0.88 with statistical significance in each model. Applying an $\omega_{1}$ = 3% lower limit of pre‐SOH RV55 Gy and an $\omega_{2}$ = 3.5% lower limit on change in RV55 Gy, retained 60% of patients experiencing the largest rectal dose reduction from the hydrogel. This may offer a clinically useful tool in deciding which patients should receive SOH implant given limited resources. Predictive models, nomograms, and a workflow diagram were produced for clinical management of SOH implant.

## 1 Introduction

Prostate is the most common cancer site in Canadian men excluding skin cancers, accounting for 20% of new cases each year and 10% of cancer deaths in men in 2018.^1^ There exist many options for treatment including surgery, external beam radiotherapy (EBRT), and brachytherapy. Many patients choose EBRT as their treatment option, however, EBRT may result in rectal, urinary, and sexual toxicities due to irradiation of organs at risk (OARs) such as the rectum, bladder, and penile bulb.^2^ The rectum is the dose limiting organ in prostate EBRT due to its proximity to the prostate.^3^, ^4^ Products to create space between the rectum and the prostate and thus potentially reduce rectal toxicities during radiotherapy have been suggested. One such product is the SpaceOAR© hydrogel (SOH) which is a polyethylene glycol‐based product injected transperineally between the prostate and the rectum, increasing the space between the organs. This additional space allows for sparing of the rectum from high dose, which has resulted in improved quality of life for patients receiving prostate EBRT.^5^, ^6^, ^7^, ^8^, ^9^, ^10^

SpaceOAR© hydrogel has been shown to reduce the rectal dose using both intensity‐modulated radiotherapy (IMRT)^5^, ^7^, ^10^, ^11^ and volumetric modulated arc therapy (VMAT) techniques.^8^, ^12^, ^13^ However, rectal dose reduction due to SOH is not equal in all patients.^14^ Patients with clinical risk factors such as the presence of hemorrhoids or previous abdominal surgery have been shown to receive large benefits from SOH.^15^ Hutchinson et al. performed a cost effectiveness study to include patient cost due to loss of income in addition to costs incurred due to medical intervention of acute and late side effects.^16^ Results indicated SOH incurred an additional $518 cost with 3D‐conformal radiotherapy (3D‐CRT) while reducing cost by $2640 when treating with high‐dose stereotactic body radiotherapy (SBRT) or stereotactic ablative radiotherapy (SABR). Additional cost effectiveness models have cited a large range in cost differential of SOH implementation.^16^, ^17^ Ensuring optimal management of SOH will allow for the most effective use of resource.

Volume of high dose to the rectum, such as the relative volume receiving 70 Gy (RV70Gy) in 78 Gy RT prescriptions, has been correlated with increased risk of rectal toxicity.^18^, ^19^ In recent years hypofractionated regimens have become increasingly common given the results of randomized trials.^20^, ^21^ There is currently no widely accepted clinically defined equivalent of RV70 Gy for hypofractionation. However, by using the linear‐quadratic model to estimate a biological equivalent dose, adjusted slightly to account for the proportion of the total prescribed dose represented by the V70 Gy, the V55 Gy can be generated as a V70Gy approximation. Certainly, rectal doses in the range of 54.25–55.2 Gy appear clinically relevant with regard to toxicity in the setting hypofractionated regimens using 3 Gy per fraction.^22^, ^23^ The rectal V55Gy, therefore is a reasonable hypofractionated approximation of the V70 Gy, applicable to 60 Gy in 20 fraction prescriptions. The RV55 Gy represents a dose level of interest with hypofractionated prostate radiotherapy prescribed to 60 Gy in 20 fractions.

Several indicators have been correlated with rectal dose–volume and have been used in predictive models. Change in rectal volume from pre‐SOH to post‐SOH plans has been shown to be proportional to the pre‐ to postchange in rectal dose.^7^ The distance from planning treatment volume (PTV) to rectum has been used to predict the lowest achievable rectum dose in IMRT prostate cancer treatment.^24^ Finally, the overlap between the expanded PTV and the rectum has been related to reduction in rectal dose.^24^, ^25^

The primary aims of this study were to retrospectively determine the pre‐SOH CT metrics which were strongly correlated with a change in rectal dose from pre‐ to post‐SOH treatment plans and to create linear models that can be used in nomograms to determine *a priori* patients that stand to benefit the most dosimetrically from SOH implant. These models would be useful in directing patient selection and serve as a guide as to the achievable rectal dose reduction for a given patient. Secondary goal was to minimize the number of contours required on pre‐SOH CT for these prediction models in order to create simple and useful decision support tools.

## 2 Methods

Some of the methods described in this study are similar to the methods used in a previous paper.^26^ However, different analysis has been applied to the data resulting in unique results and conclusions.

### 2.A Planning data sets

Anonymized CT data set of 22 patients with SOH implant between the rectum and the prostate were selected for this institutional ethics board approved retrospective study. Patients on this study received a CT scan 30–60 min prior to the fiducial and SOH implant with a comfortably full bladder and empty rectum. Patients were instructed to void and then drink 750 ml of water within a 15 min window, 1 h prior and perform a micro‐enema 2–3 h prior to the pre‐SOH CT scan. SOH was then implanted between the rectum and prostate transperinneally via ultrasound guidance followed by implantation of three to four gold fiducial markers. A post‐SOH CT scan and a pelvic MRI were taken 1 week postimplant with patients following the same bladder and rectum instructions. The MR images (T1 FSPGR and T2 proton weighted) were registered with the post‐SOH CT scan focusing primarily on the prostate and were used to contour structures of interest, in particular the SOH. One patient was removed from the studied data set due to an empty bladder in the pre‐SOH CT scan.

### 2.B Structure sets

Contouring of the prostate and seminal vesicles was performed by three experienced genitourinary radiation oncologists. Organs at risk were contoured by experienced radiation therapists and subsequently reviewed and modified as needed by the radiation oncologists. All contours were peer reviewed by the entire BC Cancer — Victoria genitourinary radiation oncology group for quality assurance. A standardized structure set was used for each patient with the CTV, rectum, bladder, SOH, penile bulb, and femoral heads being contoured. CTV was contoured as the whole prostate gland and 1 cm of the seminal vesicles to emulate patients with intermediate risk disease. PTV was produced by adding margins of 7 mm in all directions with the exception of 5 mm in the posterior direction. The rectum was contoured from the ischial tuberosities to the rectosigmoid junction as a whole organ (rectum) and as a wall structure (3 mm inner wall structure created, RW). A second rectum wall structure was created for plan optimization as per the BC Cancer — Victoria planning guidelines (RW17.5). This is an adapted PROFIT^20^ structure which stretches 17.5 mm superiorly and inferiorly of the most extreme PTV slices while maintaining a wall structure. Similarly, three bladder structures were produced: one as a whole organ (bladder), one as a wall structure (BW), and one following BC Cancer — Victoria planning guidelines (BW17.5). SOH and CTV structures were contoured on the post‐SOH CT using the fused MR image as a guide. Penile bulb was contoured as the bulbous spongiosum below the GU diaphragm and proximal to the penile shaft. Femoral heads were contoured from the heads of the femur to the area between the greater and lesser trochanters.

### 2.C Treatment planning

Treatment planning was completed using Eclipse version 13.6 to produce hypofractionated 60 Gy in 20 fraction single‐arc VMAT plans for each CT dataset with final dose calculations being completed by the anisotropic analytical algorithm (AAA, version 11.0.31). Two VMAT treatment plans were created per patient by a single planner, one pre‐SOH, and one post‐SOH. Single‐arc VMAT treatment plans were created using inverse optimization (progressive resolution optimizer version 11.0.31) with a series of optimization objectives on various structure contours. Rectal wall (RW) was used as an optimization structure as it has been shown to most effectively reduce the rectal dose when using SOH.^26^ Similarly, optimization objectives were placed on PTV, CTV, bladder wall, penile bulb, and femoral heads. Treatment plans were deemed optimal once the plan evaluation objectives shown in Table 1 were reached with a plan normalization adjustment of less than ±0.5% following the final dose calculation. This strict limit on plan normalization adjustment compared to clinical practices (up to 5% adjustment for such hypofractionated prostate plans) was placed to reduce variation between plans due to plan normalization.

**Table 1:** Plan evaluation objectives used in VMAT and IMRT treatment planning.

Structure	Metric (cGy)	Volume
PTV	V5700	≥99%
	V6300	1.00 cc
CTV	V6000	≥99%
RW17.5	V4600	≤30%
	V3700	≤50%
BW17.5	V4600	≤30%
	V3700	≤50%
Lt Femoral head	V4300	≤2.5%
Rt Femoral head	V4300	≤2.5%
Penile Bulb	V4166	≤50%

IMRT, intensity‐modulated radiotherapy; VMAT, volumetric modulated arc therapy.

The plan evaluation objectives shown in Table 1 were taken from BC Cancer — Victoria planning procedures for hypofractionated, 60 Gy in 20 fractions, prostate radiotherapy. These goals were adopted from the PROFIT^20^ and CHHiP^21^ studies on hypofractionated prostate radiotherapy. The RW17.5 and BW17.5 structures refer to the secondary wall structure contoured in which both the RW and BW are limited to 17.5 mm superiorly and inferiorly of the final PTV slice.

### 2.D Metrics of Interest

Several pre‐SOH CT geometric descriptors were used as independent metrics in the development of multiple linear regression predictive models. Indepedent metrics used in the models were extracted from Eclipse TPS or RadOnc R package version 1.1.5 using RS and RD dicom files. Rectum, RW, PTV, and CTV volumes were all extracted from Eclipse TPS pre‐SOH plan DVH text files. An additional structure was created by a boolean operation between the PTV and the rectum resulting in the volume of rectum in the PTV (RinPTV) in Eclipse TPS prior to file export. RinPTV and CTV volumes were also normalized by dividing their volumes by the rectal volume allowing for a relative metric as opposed to an absolute metric. RadOnc R package was a useful tool in accessing further geometric information from structures.^27^ The distance between rectum and CTV was a structure of interest, as such the Hausdorff distance was measured. Hausdorff distance measures the furthest point between two 2D contours.^28^ Hausdorff distance was determined along each CT slice to produce a mean distance between rectum wall and CTV (RWtoCTV). For this measurement, the height limited rectum wall, RW17.5, was used to ensure consistency in superior/inferior direction.

**Fig. 1:**
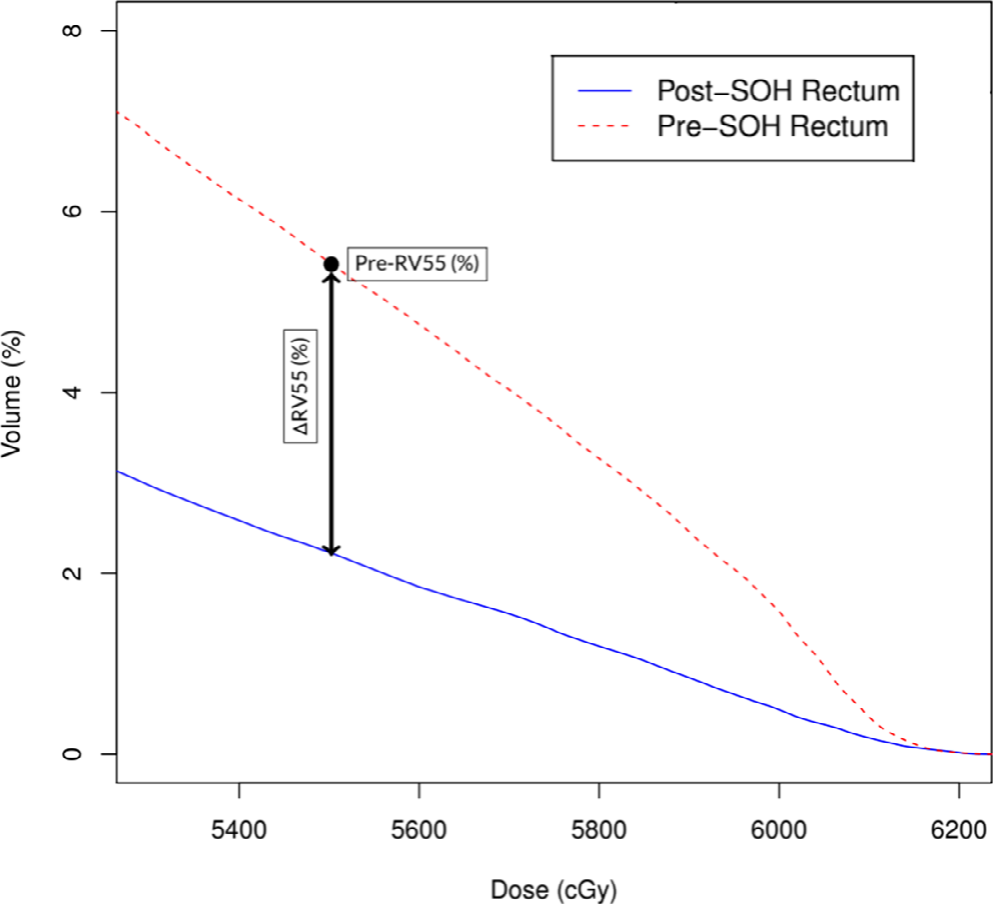
A visual representation of rectal dose metrics (ΔRV55 and Pre‐RV55) used as depedent metrics in multiple linear regression modeling. This DVH produces rectal dose metrics of ΔRV55 = 3.21% and Pre‐RV55 = 5.44%.

A number of dependent metrics were chosen for the multiple linear models and quantified. A rectal dose metric was measured in pre‐ and post‐SOH plans to determine rectal dose reduction due to SOH implant with another metric representing the pre‐SOH rectal dose. Change in RV55Gy (ΔRV55) and pre‐SOH RV55 Gy (Pre‐RV55) was used as dependent metrics in the linear models and are represented in Fig. 1.

### 2.e Statistical analysis

Modeling began by calculating Pearson correlation coefficients to identify anatomical features well correlated to dependent metrics. Independent metrics with high Pearson correlation coefficients were included in multiple linear regression models. Prior to modeling process, any independent metrics indicating dependence were identified and only one of them was selected for modeling purposes. As expected both PTV and CTV as well as rectum and rectum wall volumes were found to be dependent due to contouring methods. CTV volume was used over PTV volume as CTV represents a clinical region of interest while PTV is a geometric volume. Furthermore, contour variations are amplified when a PTV margin is applied. Rectum volume and rectum wall volume were used interchangeably, but separately.

All independent metrics were initially included in the multiple linear regression model of a selected dependent metric and then reduced through use of Akaike information criterion (AIC) and variance inflation factor (VIF). AIC represents the relative quality of the linear regression models with lower values indicating more effective models. A stepwise AIC approach was used in removing independent variables to generate an optimal linear regression model for each dependent metric (ΔRV55 and Pre‐RV55). VIF quantifies the severity of multicolinearity between variables. Independent metrics with large VIF were also removed from the model until VIF was acceptable for all variables (VIF < 5).^29^ Independent metrics chosen for multiple linear regression were associated with a beta coefficient, β. These coefficients combined with the metric creates a linear representation of the data as shown below.

(1) $$y=\beta_{1}x_{1}+\beta_{2}x_{2}+\beta_{3}x_{3}+\ldots$$

In this expression, *y* is the dependent metric, $x_{i}$ are the independent metrics used in multiple linear regression models, and $\beta_{i}$ are the beta coefficients. The relative contributions of each of these independent metrics in describing variance of the data may be described through a structure coefficient, $r_{s}$.^30^ The structure coefficient describes the contribution of independent metrics to the variance of the dependent metric.

(2) $$r_{s}=\frac{r_{\mathrm{pears}}}{\sqrt{R^{2}}}$$

The structure coefficient is described by dividing Pearson correlation coefficient, $r_{\mathrm{pears}}$, by the square root of R‐squared from the model. The squared structure coefficient, $r_{s}^{2}$, will be reported in this study.

Two models were produced per independent predictive metric: an advanced model with metrics defined using RadOnc package (Model 1) and a simpler model using metrics available in Eclipse TPS (Model 2). The selected models were evaluated using leave‐one‐out cross‐validation (LOOCV) techniques reporting the predicted R‐squared value, mean absolute error (MAE), and relative MAE (%MAE).^31^ Predicted R‐squared indicates effectiveness of model at predicting new observations while MAE represents average absolute difference between predicted and actual metrics. %MAE was reported by normalizing MAE by the range of the dependent metric. R version 3.5.3 was used for statistical analysis. Statistical tests with p‐values less than the statistical significance level of α=0.01 were deemed statistically significant.

## 3 Results

Table 2 shows the measured min, max, mean, and standard deviation of the dependent rectal dose metrics for the 21 patients used to generate predictive models while Table 3 summarizes the statistics for the independent pre‐SOH CT scan metrics. Table 3 summarizes the Pearson correlation coefficients between the dependent metrics (ΔRV55 and Pre‐RV55) and the independent variables. Pearson correlation coefficients between dependent metrics and RinPTV volume indicated strong, statistically significant, positive correlation (*R* ^2^> 0.68). Normalized RinPTV was also highly correlated with dependent metrics with correlations achieving statistical significance for ΔRV55 and Pre‐RV55. RWtoCTV led to a strong, statistically significant, negative correlation with ΔRV55 and Pre‐RV55. No other Pearson correlation coefficients showed statistically significant results.

**Table 2:** Statistical summary of the dependent metrics for the 21 patients used to model change in rectal dose after SOH implant.

Metric	Mean ± STD. Dev (Min–Max)
ΔRV55 (%)	4.5 ± 3.0 (0.8–10.8)
Pre‐RV55 (%)	5.9 ± 3.7 (0.8–13.5)

**Table 3:** Statistical summary of independent geometric variables for the 21 patients extracted from pre‐SOH CT scans using Eclipse software and RadOnc R package.

Metric	Mean ± STD. Dev (Min–Max)
Rectum Vol. (cc)	79.2 ± 32.1 (35.5–149.7)
Rectal Wall Vol. (cc)	35.5 ± 9.7 (20.5–57.8)
CTV Vol. (cc)	39.9 ± 17.2 (24.6–91.5)
Normalized CTV Vol. (%)	62.3 ± 51.7 (19.6–257.7)
RinPTV Vol. (cc)	2.4 ± 1.5 (0.1–5.3)
Normalized RinPTV Vol. (%)	3.4 ± 2.4 (0.2–10.4)
RWtoCTV (cm)	2.18 ± 0.26 (1.71–2.63)
RWtoCTV Cubed (cc)	10.36 ± 4.06 (4.99‐18.21)

### 3.A **Change in RV55Gy (** Δ **RV55) Models**

**Fig. 2:**
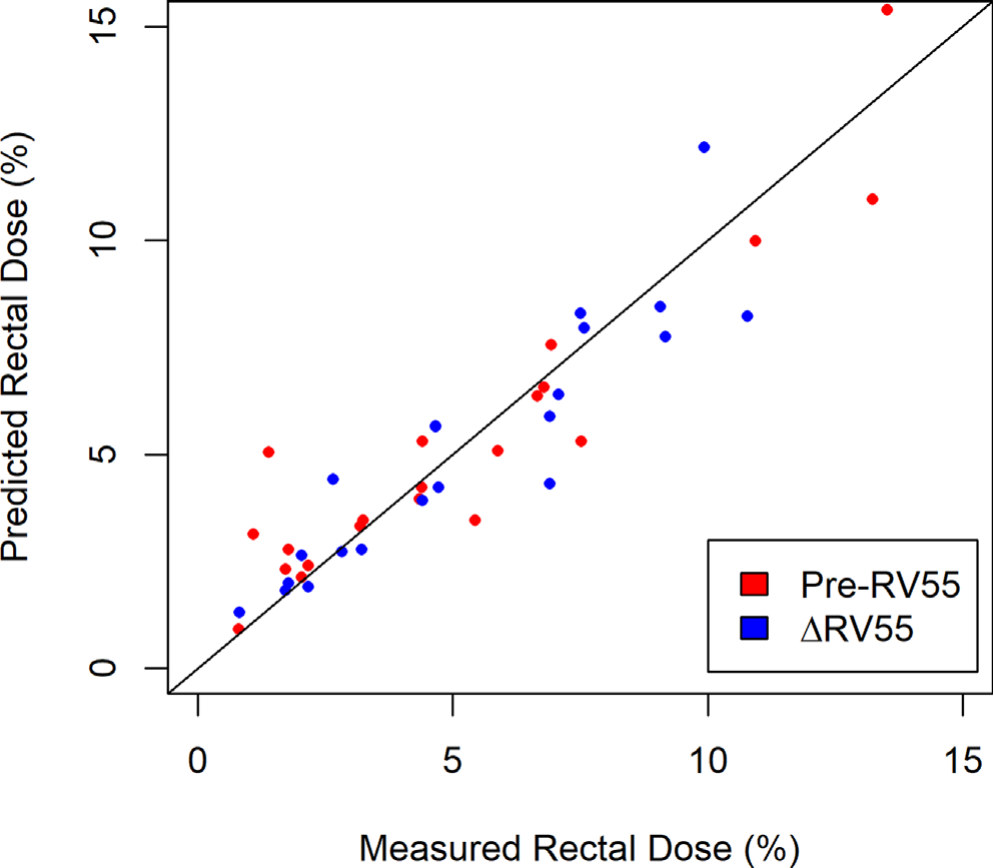
Predicted and measured RV55 for both the Pre‐RV55 and ΔRV55 models with a trendline (m=1) indicating the effectiveness of the predictive models.

Change in RV55Gy (ΔRV55), a relative dose–volume metric, was measured to be 4.5 ± 3.0 % with a range between 0.8% and 10.8% for the 21 patients selected demonstrating large variations in rectal dose sparing using SOH. ΔRV55 was predicted using normalized RinPTV, rectum volume, and RWtoCTV for Model 1 (*P* < 0.0001, *R* ^2^=0.83) and with only normalized RinPTV volume for Model 2 (*P* < 0.0001, *R* ^2^=0.81). Model coefficients are summarized in Table 4 with Fig. 2 presenting the predicted plotted against the measured values. Normalization of RinPTV volume produced higher Pearson correlation coefficients compared to RinPTV volume metric resulting in a metric with structure coefficients of about 1.00. ΔRV55 on its own does not offer enough information to make clinical decisions and must be combined with pre‐SOH dose information.

**Table 4:** Pearson correlation coefficients between dependent metrics and independent variables. Bold entries represent statistical significance.

Metrics	Pearson Coefficients	
	ΔRV55 (%)	Pre‐RV55 (%)
Rectum Vol. (cc)	−0.38	−0.33
Rectum Wall Vol. (cc)	−0.37	−0.32
RinPTV Vol. (cc)	**0.68**	**0.76**
Normalized RinPTV Vol. (%)	**0.90**	**0.92**
CTV Vol. (cc)	0.22	0.27
Normalized CTV Vol. (%)	0.29	0.32
RW to CTV Dist. (cm)	−**0.56**	−**0.61**
RW to CTV Dist. Inv. Cubed (cc^−1^)	0.33	**0.54**

### 3.B Pre‐SOH RV55Gy (Pre‐RV55) Models

Pre‐SOH RV55 Gy (Pre‐RV55) models may offer insight to relative change in rectal dose, a metric often cited in SOH papers. Pre‐RV55 was measured to be 5.9 ± 3.7% on average and ranged from 0.8% to 13.5% for the 21 patients included in the modeling. Pre‐RV55 was predicted with the use of many metrics, including rectum or RW, CTV, and RinPTV volumes (Table 5). The distance between RW and CTV was cubed and produced an effective correlation for Model 1 (*P* < 0.0001, $R^{2}$ = 0.87) but was omitted for Model 2 which only includes RW and RinPTV volumes as predictors (*P* < 0.0001, $R^{2}$ = 0.80) as summarized in Table 7. Fig. 2 presents the predicted and measured values with line of slope m = 1 indicating the optimal line of best fit. RinPTV contributed most to Model 1 and Model 2 with structure coefficients of 0.66 and 0.71, respectively.

**Table 5:** Beta, $\beta_{i}$, and squared structure, $r_{s}^{2}$, coefficients for models predicting change in RV55Gy (ΔRV55).

Metrics	ΔRV55 Models			
	Model 1		Model 2	
	β	r_s_^2^	β	r_s_^2^
Constant	−4.26 ± 4.09	–	0.73 ± 0.51	–
Normalized RinPTV Vol. (%)	1.25 ± 0.18	0.97	1.10 ± 0.12	1.00
RW to CTV (cm)	0.23 ± 0.17	0.38	–	–
Rectum Vol. (cc)	−0.0077 ± 0.0101	0.18	–	–

### 3.C Leave‐One‐Out Cross‐Validation (LOOCV)

Results from LOOCV analysis resulted in predicted R‐squared, MAE and %MAE values for each model as reported in Table 6. The smallest difference between predicted R‐squared and measured R‐squared was seen for ΔRV55 Model 2 (measured = 0.81, predicted = 0.76) while the highest predicted R‐squared (0.79) was found for Pre‐RV55 Model 2. %MAE varied from a minimum of 9.60% for ΔRV55 Model 2 to a maximum of 12.90% for ΔRV55 Model 1. %MAE for Pre‐RV55 models fell between these two values.

**Table 6:** Results from leave‐one‐out cross‐validation (LOOCV) statistical test to examine effectiveness of chosen models. Mean average error (MAE) and relative mean average error (%MAE) were reported.

Model	Predicted R‐squared	MAE	%MAE
ΔRV55 Model 1 (%)	0.63	1.27	12.90
ΔRV55 Model 2 (%)	0.76	0.96	9.60
Pre‐RV55 Model 1 (%)	0.76	1.48	11.62
Pre‐RV55 Model 2 (%)	0.79	1.35	10.65

## 4 Discussion

This study sought to produce predictive models to inform on effectiveness of SOH implant regarding rectal dose sparing based on data from 21 patients. Given its associated costs, radiation oncologists must wisely manage this new resource. A robust prediction model would be valuable in this regard to aid in optimal patient selection for SOH use. Furthermore, predictive models of rectal dose reduction can serve to guide dosimetrists as to the degree of rectal sparing that should be achievable for a given patient with SOH during the planning process. Rectal toxicity may be linked to metrics quantifying high dose (ΔRV55 and Pre‐RV55) which were used as dependent dose metrics in the linear models. Independent pre‐SOH volume metrics of interest were selected based on previous studies and Pearson correlation coefficients obtained during the modeling process. Pearson correlation coefficients indicated that volume of RinPTV (absolute and normalized) and RWtoCTV distance (absolute and inverse cubed) provided the highest correlation to dependent rectal dose metrics (Table 2). These findings are in line with other studies. More specifically, Wang et al.^24^ found the overlap between PTV and rectum was related to reduction in rectal dose and the distance from PTV to the rectum was used to predict the lowest achievable rectal dose. Additionally, Mariados et al.^5^ showed that the change in rectum volume from pre‐ to post‐SOH plans was proportional to the change in rectal dose. In this retrospective study, rectal volume metrics such as rectum or RW volumes were also correlated with the change in rectal dose metrics (Table 2).

The stepwise approach of linear regression modeling using AIC and VIF for model selection retained a variety of independent variables for each dependent metric. When modeling, there is always a risk of overfitting a model — describing the random error in the data instead of the relationship between variables — when too many independent variables are used in the model causing an inflated R‐squared value.^31^ In an attempt to combat this effect, a maximum of three independent variables were used to describe the dependent metrics given the limited number (n = 21) of datapoints. Two models were produced for each dependent metric: Model 1 and Model 2. Model 1 represented the best fit with metrics available using both RadOnc R package and Eclipse TPS. RadOnc R package metrics require additional computation time making them less clinically practical where decisions must often be made promptly. As such, Model 2 was developed without application of RadOnc metrics using only volumes available from the treatment planning software.

All four generated models were evaluated using LOOCV which removed a single sample from the model and reperformed the linear regression with the new predicted value compared to the measured value for all 21 datasets. The difference in measured and predicted values was averaged over all datasets, producing a predicted R‐squared and a MAE. %MAE was reported by normalizing MAE by the range of the dependent metric. Relative MAE (%MAE) represents the uncertainty in the model after predicting a dependent rectal dose reduction metric and ranged from 9.60% to 12.90% for all models. Increasing the number of patients within the modeling process should decrease the %MAE thus further improving the quality of these predictive models which currently have an uncertainty in the ballpark of 10–13%. While MAE and %MAE both offer insight on model uncertainty, the predicted R‐squared value provides information on the effectiveness of the model at predicting ΔRV55 and Pre‐RV55 metrics. All models had strong predicted R‐squared values (R^2^> 0.75) thus supporting their effectiveness at predicting both ΔRV55 and Pre‐RV55 metrics, however, models based on TPS variables (i.e., Model 2) had the highest predicted R‐squared values. The small difference between the predicted R‐squared and reported model R‐squared offers further evidence that the generated models are not overfitted. Specifically, the difference in predicted and reported R‐squared values was 0.05 for ΔRV55 (Predicted R^2^ = 0.76, R^2^ = 0.81) and 0.08 for Pre‐RV55 (predicted R^2^ = 0.79, R^2^ = 0.87), respectively. The high predicted R‐squared combined with the small difference in predicted and reported R‐squared values indicates these models are effective at predicting new observations while not being overfitted.

Describing rectal dose information through a single metric is difficult to accomplish. High rectal dose has been associated with increased risk of rectal toxicity^18^, ^19^ and should be encompassed in the metric of interest for assessment of rectal dose reduction associated with SOH implant. RV70Gy has been a commonly used metric in prostate radiotherapy plan evaluation for 78 Gy prescriptions delivered in 2Gy per fraction. A 25% decrease in RV70Gy was reported, and considered a clinically significant benefit, when radiotherapy delivery techniques changed from 3DCRT to IMRT.^32^ This metric has been used in SOH studies to evaluate the effectiveness of SOH implant for rectal dose reduction.^5^, ^7^, ^33^ Unfortunately, the widespread adoption of hypofractionation in recent years limits the applicability of RV70Gy.^34^ However, applying the linear‐quadratic model (α/β = 3), with consideration to the proportion of the total prescribed dose represented by RV70Gy, provides RV55Gy as a reasonable **c**orresponding dose approximation in the setting of hypofractionation (60Gy in 20 fractions). This metric provides a clinically intuitive representation of the rectal dose, useful for characterizing the change in rectal dose from pre‐ to post‐SOH implant.

ΔRV55 was found to be highly correlated with independent metrics such as RinPTV, normalized RinPTV, and RWtoCTV. RWtoCTV ($r_{\mathrm{pears}}=-0.56$) offered a higher correlation compared to inverse cubed RWtoCTV metric ($r_{\mathrm{pears}}=0.33$) while the normalized RinPTV offered a higher correlation compared to absolute RinPTV ($r_{\mathrm{pears}}=0.90$), likely due to the relative nature of ΔRV55. Through the modeling process, normalized RinPTV was found to contribute the most to ΔRV55 as indicated by the large structure coefficient for both Model 1 and Model 2 ($r_{s}^{2}=0.97$). Linear fit results for ΔRV55 Model 1 and Model 2 presented in Table 8 are associated with strong R‐squared values of 0.83 and 0.81, respectively. The measured and predicted values presented in Fig. 2 are well distributed to either side of the ideal slope m = 1 line. In addition, %MAE for each model is approximately 10%. Given the strong correlation values, *P*‐values < 0.0001 and low error, ΔRV55 can be effectively predicted via TPS‐based independent metrics.

Similar to ΔRV55, Pre‐RV55 was highly correlated with RinPTV and RWtoCTV. As shown in Table 7, both these metrics resulted in the highest structure coefficients for both Model 1 and Model 2. Both Pre‐RV55 Models 1 and 2 yielded larger R‐squared values (0.88 and 0.87, respectively) compared to the ΔRV55 Model 1 and Model 2 (0.83 and 0.81, respectively). The predicted R‐squared values from LOOCV for Pre‐RV55 were found to be slightly larger than for ΔRV55 at 0.76 and 0.79, indicating that these models are also able to effectively predict new observations. The measured and predicted values are also presented in Fig. 2 and lie near the ideal trendline. However, points with a low Pre‐RV55 value seem to be slightly overpredicted while values lying near the center of the distribution are well predicted. LOOCV resulted in a %MAE of 11.62% for Model 1 and 10.65% for Model 2 which lie near the values reported for Δ RV55. Due to the low %MAE and high predicted R‐squared value, both models can effectively predict Pre‐RV55.

**Table 7:** Beta, $\beta_{i}$, and squared structure, $r_{s}^{2}$, coefficients for models predicting pre‐SOH RV55Gy (Pre‐RV55).

Metrics	Pre‐RV55 Models			
	Model 1		Model 2	
	β	r_s_^2^	β	r_s_^2^
Constant	−0.45 ± 1.10	–	0.67 ± 0.61	–
Normalized RinPTV Vol. (cc)	1.19 ± 0.20	0.97	1.37 ± 0.13	0.98
Normalized CTV Vol. (cc)	0.012 ± 0.007	0.12	0.009 ± 0.006	0.12
RW to CTV Inv. Cube (cc^−1^)	13.88 ± 11.4	0.33	–	–

**Fig. 3:**
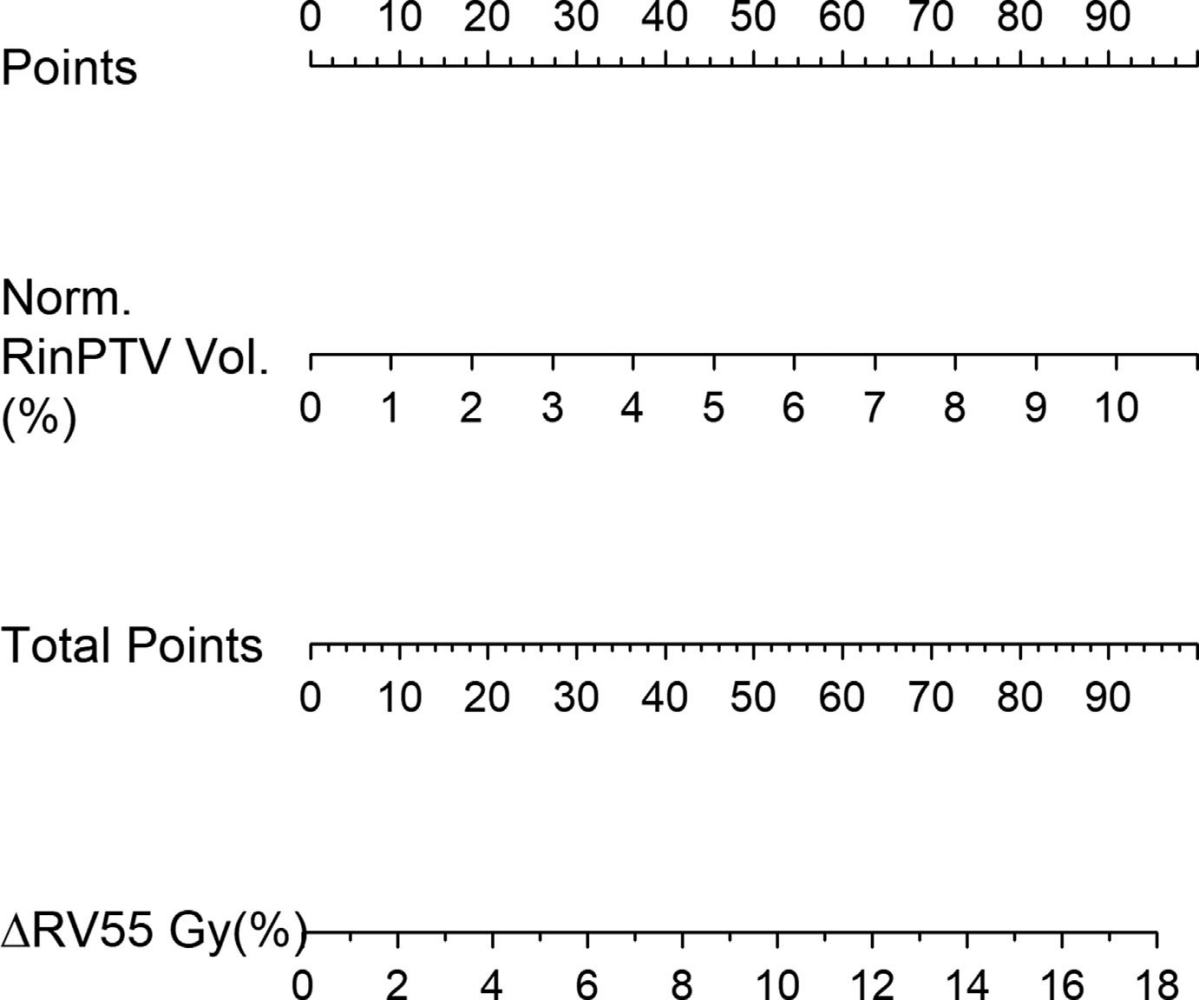
Nomogram prepared from change in RV55 Gy Model 2. The points contributed from the normalized RinPTV volume will correlate with the change in RV55 Gy metric as per model.

**Fig. 4:**
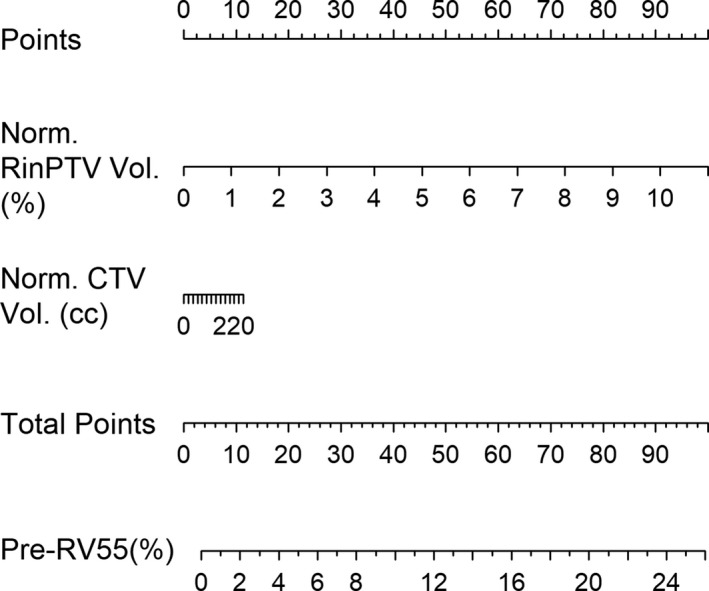
Nomogram prepared from pre‐SOH RV55 Gy Model 2. Normalized CTV and RinPTV volumes contribute to the model, from which the pre‐SOH RV55 Gy metric can be predicted. SOH, SpaceOAR© hydrogel.

**Fig. 5:**
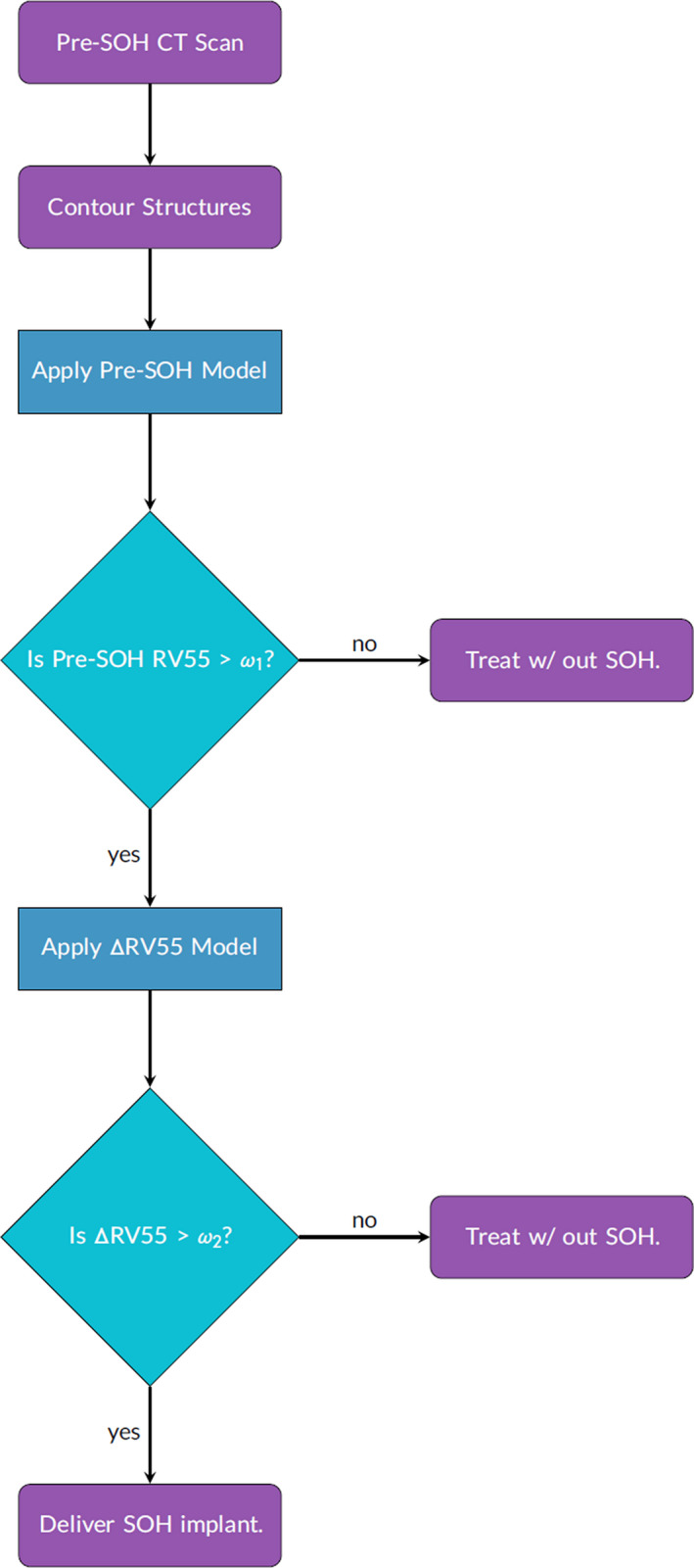
Decision‐making flowchart applying pre‐SOH RV55 Gy and ΔRV55 models. Limits of $\omega_{1}$ and $\omega_{2}$ may be employed on the pre‐SOH RV55 and ΔRV55 respectively to ensure patients receiving the implant experience a large reduction in rectal dose. SOH, SpaceOAR© hydrogel.

Producing clinically useful decision support tools for SOH management was a priority when developing these predictive models. In both ΔRV55 and Pre‐RV55 models, Model 2 resulted in a lower %MAE and higher predicted R‐squared as shown by Table 8. Model 2 independent variables are also more clinically accessible, requiring only a treatment planning system. Nomograms for Δ RV55 and Pre‐RV55 were produced using Model 2 and are shown in Figs. 3, 4. To guide radiation oncologists to select *a priori* patients who stand to benefit most dosimetrically from SOH implant, a decision flowchart (Fig. 5) was created based on the above models and governed by cutoff limits $\omega_{1}$ for Pre‐RV55 and $\omega_{2}$ for ΔRV55.

**Table 8:** Summary of *P*‐value, R‐squared and MAE from LOOCV for each linear regression model presented.

Model	P‐value	R‐squared	Predicted R‐squared	MAE	%MAE
ΔRV55 Model 1 (%)	*P* < 0.0001	0.83	0.63	1.29	12.90
ΔRV55 Model 2 (%)	*P* < 0.0001	0.81	0.76	0.96	9.60
Pre‐RV55 Model 1 (%)	*P* < 0.0001	0.88	0.76	1.48	11.62
Pre‐RV55 Model 2 (%)	*P* < 0.0001	0.87	0.79	1.35	10.65

LOOCV, leave‐one‐out cross‐validation; MAE, mean absolute error.

Cutoff limits for both Pre‐RV55 and ΔRV55 should be governed by published data on SOH‐based rectal sparing as well as local variations in SOH‐based radiotherapy planning and delivery. According to the multi‐institutional SOH pivotal trial by Mariados et al., the mean post‐SOH RV70Gy achieved for this large group of patients was 3.3 ± 3.2% while the pre‐SOH and mean change in RV70Gy were 12.4 ± 5.4% and 9.1 ± 8.6%, respectively. In light of the low toxicity observed in the SOH cohort of Mariados et al, a Pre‐RV55 cutoff limit of 3.0% is proposed as patients with Pre‐RV55 below this value are less likely to develop bowel toxicities. Using $\omega_{1}$ = 3.0% in the SOH decision‐making flowchart (Fig. 5), 16/21 patients were retained in the implant pool. Given the observed differences in Pre‐RV55 and ΔRV55 between Mariados et al. and our single institution cohort a ΔRV55 cutoff limit of $\omega_{2}$ = 3.5% is proposed for institutions that have planning strategies similar to those described in Paetkau et al.^24^ Applying $\omega_{2}$ = 3.5% in the decision‐making flowchart removed an additional four patients therefore identifying 12/21 patients that are likely to benefit the most dosimetrically from an SOH implant. The patients removed from the implant pool had a mean pre‐SOH RV55 of 3.0 ± 1.9%, which lies below the RV70Gy achieved by Mariados et al., while the remaining patients in the implant pool had a mean post‐SOH RV55 of 1.8 ± 1.7%. No statistically significant differences were found between these two sub‐population as indicated by the student's t‐test p‐value = 0.18 for mean volumes.

**Fig. 6:**
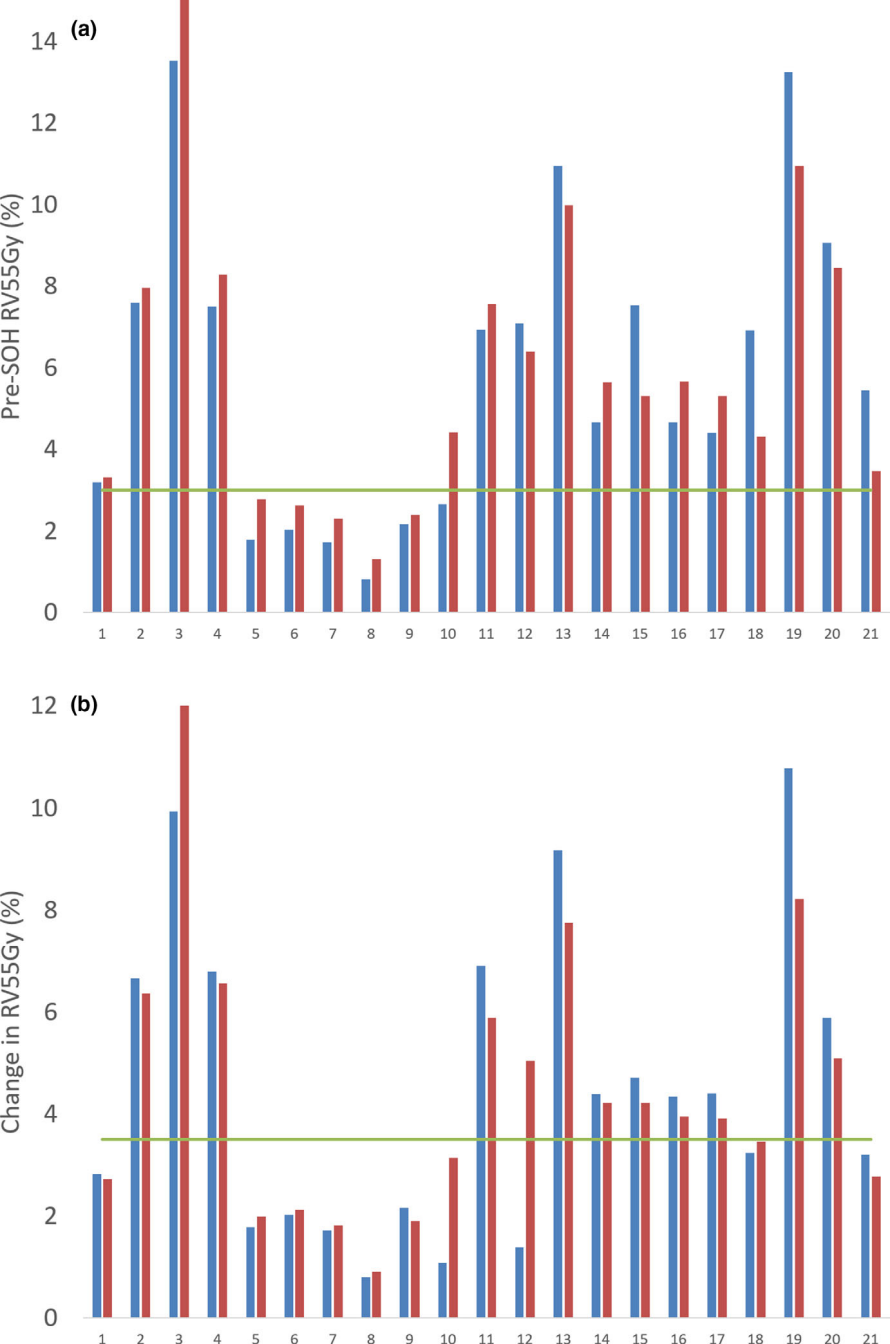
Histogram of measured and predicted (a) pre‐SOH RV55Gy and (b) ΔRV55Gy for each patient along with proposed cutoff limits. SOH, SpaceOAR© hydrogel.

The flowchart based on the above prediction models and associated cutoff limits removed a total of eight patients from the implant pool. The above cutoff limits are based on our single institution results and adjustments to these limits will remove greater or fewer patients from the implant pool. Due to the imperfection of the prediction models, one patient was incorrectly kept in the implant pool during the first selection step but correctly removed during the second step, and one patient was incorrectly removed during the second selection stage. These selection errors may be observed in the paired (measured–predicted) patient data sets shown in Fig. 6. Patients with which the predictive value lay above the cutoff while measured was below or vice versa were incorrectly selected. This is due to the imperfection of the models as quantified by the %MAE, and as such when a value near the limit is predicted, there may be errors in the patient selection process.

Current proposed cutoff limits on Pre‐RV55 and ΔRV55 metrics would result in about 60% of prostate patients receiving SOH implant. This retention rate aligns well economically with the resources available at BC Cancer — Victoria to implement the SOH implant. Additionally, there is significant patient benefit to these prediction models. It is important to consider that a small reduction in rectal dose may not be significant enough to put a patient through the discomfort (i.e., mild pain) and potential complications (i.e., low risk of sepsis) of the procedure. These predictive models and proposed cutoff limits aid in this patient selection.

The models derived in this study may offer effective SOH management guidelines, however, their limitations must be considered. The maximum ΔRV55 from pre‐ to post‐SOH observed was approximately 10% (Table 3) and as such any value predicted above this value may have a large associated error. Limits could be applied to independent geometric variables (Table 3), specifically RinPTV, but this would limit the application of the model. For patients with no PTV overlapping the rectum, many of these models will not be applicable. However, this could only occur in a patient with favorable rectal/perirectal anatomy in the setting of a precision RT technique (i.e., SABR with implanted transponders or robotic delivery) with a narrow posterior margin (i.e., 2‐3mm). Additionally, models were built using dosimetric information from plans where a specific, but commonly utilized, PTV margin was applied. PTV margins depend heavily on the type of image‐guidance applied.^35^, ^36^ Other PTV margins may change RinPTV metrics along with RV55Gy dose. Reducing PTV margins would reduce RinPTV, which subsequently reduces predicted Pre‐RV55 and change in rectal dose metrics. Metrics collected in this study were measured using specific guidelines. Varying these guidelines would result in changes to linear regression correlations and coefficients. Institutions that wish to adopt these models and decision flowchart for SOH management are encouraged to validate these prediction models or generate their own prediction models using the variables identified in this study and establish cutoff limits based on their clinical experience with SOH.

Linear regression models for rectal dose prediction have been produced by Pinkawa et al., Yang et al. and Hwang et al.^37^, ^38^, ^39^. Yang et al. employed overlap‐volume histogram metrics while Hwang et al. correlated hydrogel placement and perirectal space creation to predict rectal dosimetry. The tool produced by Hwang et al. was used to evaluate hydrogel placement as a learning tool to reduce learning curve seen in hydrogel implant. Models were produced based on post‐SOH CT scans with distance from CTV to rectum as a primary metric to predict rectal dosimetric endpoints. The definition of distance between CTV and rectum was different compared to the study presented here but the use of a similar metric between models is significant. A pre‐SOH decision model study was completed by van Wijk et al.^14^ which produced a virtual spacer implant model. This simulated an implantable rectal spacer (IRS) to help identify patients in which IRS was not beneficial. This study offered a decision support tool which provides cost effectiveness analysis along with toxicity prediction. Models presented here predicted change in rectal dose using nomograms after a single pre‐SOH CT scan and contouring of rectum and CTV structures. Implementing these linear models may require an additional CT scan prior to SOH implant if one was not completed during diagnosis and staging. This additional step in patient process increases dose, requires further resources for contouring, CT scan scheduling and may be subject to error from bladder and bowel preparation methods.

Identifying patients that would benefit dosimetrically earlier in the process would further aid in decision‐making for both doctors and patients. Applying these models to diagnostic CT scans would improve the workflow of SOH management described in this study. Such a task may be performed with radiomics packages available in languages such as R and Python. The present models may potentially be applied to a diagnostic CT scan if the appropriate bowel and bladder preparation is performed prior to imaging.

## 5 Conclusions

Predictive models were created for change in RV55 Gy (ΔRV55) and pre‐SOH RV55 Gy (Pre‐RV55). All models reached statistical significance with ΔRV55 and Pre‐RV55 models reaching *R* ^2^ and predicted *R* ^2^ values greater than 0.75 and MAE between 9.6–12.9%. Volumes of rectum in PTV (RinPTV), as well as CTV and rectum volumes offered highest correlation with dependent metrics. Distance between rectal wall and CTV contours offered high Pearson correlation but was not included in TPS‐based models (i.e., Model 2). Applying a lower limit of $\omega_{1}$ = 3% on the Pre‐RV55 model and $\omega_{2}$ = 3.5% on ΔRV55 models retained approximately 60% patients receiving sufficient rectal dose reduction from the SOH implant (12/21). The mean post‐SOH RV55 Gy for the retained patents (1.8 ± 1.7%) was found not to be statistically different from the mean pre‐SOH RV55 Gy of those not retained (3.0 ± 1.9%) in the implant process. Linear predictive models along with specific limits offer decision support tools for more effective SOH implant management and may aid in the planning process.

## Conflict of Interest

No conflict of interest.
